# Life After Kidney Transplantation: The Time for a New Narrative

**DOI:** 10.3389/ti.2025.14074

**Published:** 2025-05-12

**Authors:** Kevin John Fowler

**Affiliations:** The Voice of the Patient, Saint Louis, MO, United States

**Keywords:** kidney transplant, patient reported outcome measures, patient advocacy, life participation, post kidney transplant

## Abstract

The first successful kidney transplant in December 1954 between the Herrick brothers ushered in a new field of medicine. Over the almost seventy years, thousands of lives have been saved and patient survival has improved. There is one area of kidney transplant patient care that has been overlooked. Patient quality and ability to participate in life have not been adequately studied. This is due in part to the false narrative of life after kidney transplantation. The false narrative has developed due to the patient voice not being heard due to a variety of factors. The development and implementation of Patient Reported Outcome Measures into clinical practice and clinical trials is the first step ensuring the patient voice is heard systematically. By enabling the patient voice to be heard, I hope this result in a new narrative that is patient centered.

## Introduction

When I was contacted to serve as a co-editor, I was thrilled for the opportunity to contribute. For myself and for many other kidney transplant recipients, we only truly feel understood by fellow recipients. The lack of understanding by some members of the medical community and sometimes our loved ones can create a sense of isolation and loneliness during different parts of our journeys. The objective of this issue of Transplant International is to provide similar insights for the transplant community. It is my hope that these insights will be acted upon so that systemic changes are made in the way transplant care is provided. Before change can be initiated, the argument for change must be articulated through an honest narrative on life after kidney transplantation. Through my personal and professional experience, the objective of this article is to educate this audience why a new narrative for life after kidney transplantation is needed.

## Life Before Transplantation

Prior to my kidney failure, I was seen by a primary care physician. Since my mother had Autosomal Dominant Polycystic Kidney Disease (ADPKD), I was aware that I had a 50% chance of inheriting her medical condition. After witnessing my mom suffer on hemodialysis and die at 52, I lived in fear that I would suffer the same fate as her. My primary care physician monitored my kidney function for any signs of changes in kidney function. According to my physician, my kidney function was normal, and I had nothing to be concerned about in the near-term future. Later that year after my annual appointment, I experienced back pain during the Holiday Season. Initially, I attributed it to firewood that I had picked up. Since flank back pain can be a symptom of ADPKD, I decided to determine if I had the same disease. I contacted my doctor and requested an ultrasound test. This test would determine definitively whether I had ADPKD.

The test confirmed that I had inherited ADPKD. In contrast to the earlier optimistic prognosis, my doctor informed me that I would be in kidney failure in 3–5 years. He informed me that I would either start dialysis or have a kidney transplant in the time frame provided. I instinctively and immediately declined the offer. The doctor that said my kidney function was fine was now making a nephrology referral. I trusted my intuition that I needed to advocate for myself. Through my self-advocacy I was able to receive the best treatment for kidney failure. My ability to trust my instincts enabled me to seek the best treatment option, and to live my best life afterwards.

I called a physician friend at an Academic Medical Center requesting his recommendation for a nephrology referral. He recommended a colleague, and I scheduled my appointment with the nephrologist. On the first appointment, my doctor informed me that I was a good candidate to receive a pre-emptive kidney transplant. This treatment would provide the best long-term outcomes for End Stage Kidney Disease (ESKD) while also avoiding dialysis. While I was overjoyed to learn this information, I was left wondering why this was the first time I was learning about this treatment option.

As I came to learn later, in the United States (US) only 3.5% [1] of incident kidney failure patients receive a pre-emptive kidney transplant. This is because most patients with kidney disease are unaware of their condition. In fact, approximately 40% of kidney failure patients crash into dialysis. In other words, these patients are unaware that they are progressing into kidney failure until dialysis is administered to save their lives. When the US Medicare End Stage Renal Disease benefit was passed into law in 1973 [2], it eliminated the need to ration dialysis treatment. While it assured that ESKD patients would have access to dialysis treatment, it failed to evolve over time until the Advancing American Kidney Health Executive Order was issued in 2019 [3].

As my kidney function continued to decline, my nephrologist created an environment for me to develop self-management skills such as understanding my lab values, and how to prepare for the day when I would receive a kidney transplant. There is one area that stands out among all aspects of my patient care. He explained the cardiovascular risks post kidney transplant, and educated me on the benefits of routine exercise to prepare for my kidney transplant. I started doing an elliptical machine workout three to five days weekly. This habit helped me to manage an uncertain future. The decision to exercise was something within my sphere of control. I would have thoughts constantly of whether I would be able to find a donor or whether my kidney transplant would be successful. The decision to choose to exercise was an act of centering in the present moment and helped to quiet my anxious thoughts. Later, I learned that it was not a common practice for nephrologists to educate their patients on the benefits of exercise [4].

## Transitioning to a New Life

The first-year post-transplant was one of immense gratitude while adjusting to a new life. The sense of joy and profound gratitude can never be adequately described. The selfless act of my living donor combined with the support that I received from my wife and community was so powerful that I knew my life was changed permanently. Amidst this sense of awe and wonderment, there were aspects of my new life that I had not been adequately educated upon prior to my transplant.

The first challenge was psychological. I was 43 years old, and during that first year, I struggled with my own mortality. Our children were very young, and I ruminated constantly on whether I would be around to see them grow up. When I had my follow up appointments with my transplant team, the discussion always centered upon lab values, ensuring I was taking my transplant medications, etc. There was never any discussion about how I was adapting to my new life with chronic immunosuppression. I clearly sensed there was an implied message that I should be grateful for my kidney, and that I should get on with my life.

The second challenge was adjusting to my kidney transplant medications. Within a year after my kidney transplant, I began to experience cognitive issues and fogginess. When I reported this to my transplant team, my experience was not validated. Rather, they stated that this condition was not caused by my medications. The conversation shifted to reminding me that I should be grateful to have received a pre-emptive kidney transplant. Contrary to what my transplant team was telling me, I knew something was not right. I was looking for a member of my care team to validate my experience, and no one did. The psychological and medication challenges combined with the pressures of a job promotion became too much for me to handle. Eventually, I experienced deep depression. I could not find any answers to my questions. Frustrated at not feeling understood with diminished quality of life, I drew upon the lessons of my pre-transplant nephrologist.

## Weathering the Storms Post-Transplant

I remembered the lessons on the benefits of exercise. I started exercising to regain some control back in my life. After I exercised, I would feel better not only physically but mentally. The physical activity resulted in improved cerebral blood flow to my brain. Routine exercise became a staple in my life because of its ability to counteract the side effects of tacrolimus. I took this action out of desperation to feel better. I needed to feel better so that I could perform my job. I started journal writing, and it provided the benefit of re-framing my new life. Rather than ruminating on thoughts on the length of my life, I shifted my efforts to towards empowering myself by taking acting action over what was in my sphere of control. I had control over whether I exercised, prepared for my medical appointments, etc. I read a book on the history of kidney transplantation, “The Puzzle People” [5], and this book made a lasting impression upon me. In the book, transplant recipients shared that they probably would never have made their life achievements without having a kidney transplant. The kidney transplant experience provided them with a greater sense of purpose with the awareness of the brevity of life. The decision to choose how I wanted to frame my life empowered me to move forward. For example, I framed my own experience as an example for our children to learn from. Over time, I added meditation to my routine of vigorous exercise and journal writing. In turn, the net benefit of my discipline was that I created a ballast to manage the ongoing challenges of managing a chronic disease.

The set of challenges after transplant have been constant and unremitting. There have been multiple hospitalizations due to infections. Once hospitalized, there was the challenge of ensuring physicians were not using nephrotoxic medications or the risk of having an acute kidney injury. There have been multiple MOHs surgeries due to squamous cell carcinoma and melanoma cancer. I have had a radical prostatectomy and three covid episodes. On top of all of that, there has been the constant challenge of managing the never-ending ups and downs of lab results while ensuring I have access to my transplant medications and access to health insurance. In the back of my mind, there is always the constant worry of my kidney failing. My routine of discipline has prepared me to effectively manage the ongoing challenges.

## Countering the Established Narrative for Kidney Transplantation

Prior to my kidney transplant, I was led to believe that everything would be ok once I received my new kidney. While it is true that my health was restored, the adjustment post-transplant was something that I was not prepared for. I was under the impression that I would have my kidney transplant, and my life would return to normal. This perception was formed in part by my kidney transplant team, and the narrative surrounding kidney transplantation. Before my transplant, I read all the celebratory stories of organ donation and kidney transplantation. No where in these stories was there any acknowledgement about some of difficulties experienced due to chronic immunosupression, or guidance to overcome the challenges. This perception has only been reinforced by published studies that tout improvement in kidney transplant survival without addressing quality of life [6].

Upon closer examination the established narrative of transplantation is contrary to published evidence. In response to a request from the Social Security Administration the National Academies of Science, Engineering, and Medicine conducted a day and a half meeting on *Organ Transplantation and Disability: A Workshop* [7]. The workshop included presentations on the functional outcomes for individuals who are recipients of organ transplants: including those of the kidney, heart liver, and lung.

The evidence presented contrasted sharply with the public perception of transplantation. Employment the first year after transplantation was quite low for all organ recipients. For kidney, liver, heart, and lung it was 31%, 21%, 21% and 14% respectively. The causes of low employment are multifactorial: lack of physical rehabilitation, side effects of transplant medications, depression, absence of patient reported outcome measures (PROMs), etc. PROMs would play a key role in enabling transplant recipients to acknowledge what they are feeling. Recently, Allison Jaure PhD published in Kidney International *Validation of a Core Patient Reported Outcome Measure for Kidney Transplant Recipients: the SONG Life Participation Instrument* [8]. The novel instrument measured activities that are important to patients such as ability to work and participate in family activities. In other words, it measures the value of the kidney transplant to the recipient. When I assess the value of my kidney transplant, I measure its value in being able to work the entire time, put my children through college, and see them grow into young adults.

The process to improve life after transplants starts with a baseline assessment of the population. To that end, the American Society of Transplantation has issued a comprehensive patient survey for all transplant recipients on a broad number of domains. One of the domains is patient reported quality of life. When the results are published, it should provide additional support for the implementation of PROMs into clinical practice. My personal example of life post-transplant and low employment for solid organ transplant recipients are just two aspects that counter the prevailing narrative of life after transplant. It is difficult to advance the field of transplantation if payors, regulators, policymakers, and patients themselves do not perceive the need for improvement. I have provided three recommendations to begin the process of initiating systemic change bu.

### Patient Reported Outcome Measures (PROMs)

As I have described above, there Is a need to implement PROMs into clinical practice. The adoption of PROMs would initiate the process for the patient voice to be heard and serve as a catalyst for patient engagement. In turn, patients would be directed to interventions to improve quality of life while additional research and resources should be increased to improving quality of life and employment. The inclusions of PROMs in pharmaceutical and device manufacturer clinical trials may offer also offer a path to differentiation in treatments and a path to regulatory approval.

### New Care Model Pilots

In the US, the CMS Innovation (CMMI) Center has introduced value-based care models to incentivize increased use of home dialysis and kidney transplantation. With this as a precedent, CMMI can address the lack of rehabilitation post kidney transplantation. As a starting point, a pilot model that offers physical rehabilitation, psychological counseling, mentorship, etc. Would be a step forward in validating the patient experience while aligning with national policy. In the US expanding kidney transplant access and the volume of kidney transplants have been formalized through reform of the Organ Procurement Organizations. While this is a positive achievement, the full value of a kidney transplant will not be achieved without rehabilitation support. It is analogous to buying an expensive automobile without routine service.

### Elevation of the Patient Voice With Regulatory Agencies

In recent years, the American Society of Transplantation (AST) and the European Society of Transplantation (ESOT) have taken meaningful action to elevate the patient voice in their professional societies. The AST has formed the Transplant Advisory Council, and ESOT has formed the ESOT-European Transplant Patients Organization Alliance that has resulted in patient representation in meetings and workgroups. Through my experience in patient advocacy, I have observed a lack of alignment and coordination between regulatory agencies regarding kidney transplantation. For example, in November 2023 I attended the FDA Public Workshop on “Endpoints and Trial Designs to Advance Drug Development in Kidney Transplantation.” Considering that the Social Security Administration conducted a workshop on organ donation and disability, their attendance at the meeting would have educated them on how the side effects and health risks of transplant medications can contribute to the difficulty of returning to work. My request is for AST and ESOT Is to leverage their patient councils to educate a broad number of regulatory agencies on the patient experience to gain a holistic understanding of the patient journey, and the unmet needs in kidney transplantation.

On December 23 2024, it was the 70th anniversary of the first successful kidney transplant. This extraordinary scientific and medical innovation has added life years to thousands of patients globally. It is now time to build upon improved patient survival and improve the quality of life of kidney transplant recipients. Acknowledging this unmet need can serve as a catalyst to engage with policymakers, regulators, Life Science companies, etc. To incentivize innovation in kidney transplantation while providing holistic patient care. I ask the global kidney transplant community to learn from the lessons of the American Society of Nephrology and the Kidney Health Initiative (KHI).

Since 2015, I have served as a Kidney Health Initiative volunteer through the Patient Family Partnership Council, and the Board of Directors. During my service, I have witnessed first-hand the transformation of nephrology patient care. The US nephrology community is in midst of changing from a system of care that financially rewarded placing patients on dialysis to one that prioritizes the detection and early intervention of kidney diseases. This change never would have happened so quickly if not for the American Society of Nephrology (ASN) elevating the patient voice as a stakeholder.

My call to action is for the global kidney transplant community to learn the ASN lessons. I would like to see the global kidney transplant community prioritize listening to the patient voice. To that end, I would like the community to focus upon the implementation of PROMS in patient care. This would ensure the patient voice is heard in a systemic manner so that improvement in quality of life is a global priority.

## Data Availability

The original contributions presented in the study are included in the article/supplementary material, further inquiries can be directed to the corresponding author.
